# Characterization of human plasma-derived exosomal RNAs by deep sequencing

**DOI:** 10.1186/1471-2164-14-319

**Published:** 2013-05-10

**Authors:** Xiaoyi Huang, Tiezheng Yuan, Michael Tschannen, Zhifu Sun, Howard Jacob, Meijun Du, Meihua Liang, Rachel L Dittmar, Yong Liu, Mingyu Liang, Manish Kohli, Stephen N Thibodeau, Lisa Boardman, Liang Wang

**Affiliations:** 1 Department of Pathology and Cancer Center, Medical College of Wisconsin, Milwaukee, WI, 53226, USA; 2 Human Molecular Genetics Center, Medical College of Wisconsin, Milwaukee, WI, 53226, USA; 3 Division of Biomedical Statistics and Informatics, Mayo Clinic, Rochester, MN, 55905, USA; 4 Department of Endocrinology, The Second Affiliated Hospital of Harbin Medical University, Harbin, 150086, China; 5 Department of Physiology, Medical College of Wisconsi, Milwaukee, WI, 53226, USA; 6 Department of Oncology, Mayo Clinic, Rochester, MN, 55905, USA; 7 Department of Laboratory Medicine and Pathology, Mayo Clinic, Rochester, MN, 55905, USA

**Keywords:** Exosome, microRNA, Next generation sequencing, Plasma, Biomarker

## Abstract

**Background:**

Exosomes, endosome-derived membrane microvesicles, contain specific RNA transcripts that are thought to be involved in cell-cell communication. These RNA transcripts have great potential as disease biomarkers. To characterize exosomal RNA profiles systemically, we performed RNA sequencing analysis using three human plasma samples and evaluated the efficacies of small RNA library preparation protocols from three manufacturers. In all we evaluated 14 libraries (7 replicates).

**Results:**

From the 14 size-selected sequencing libraries, we obtained a total of 101.8 million raw single-end reads, an average of about 7.27 million reads per library. Sequence analysis showed that there was a diverse collection of the exosomal RNA species among which microRNAs (miRNAs) were the most abundant, making up over 42.32% of all raw reads and 76.20% of all mappable reads. At the current read depth, 593 miRNAs were detectable. The five most common miRNAs (miR-99a-5p, miR-128, miR-124-3p, miR-22-3p, and miR-99b-5p) collectively accounted for 48.99% of all mappable miRNA sequences. MiRNA target gene enrichment analysis suggested that the highly abundant miRNAs may play an important role in biological functions such as protein phosphorylation, RNA splicing, chromosomal abnormality, and angiogenesis. From the unknown RNA sequences, we predicted 185 potential miRNA candidates. Furthermore, we detected significant fractions of other RNA species including ribosomal RNA (9.16% of all mappable counts), long non-coding RNA (3.36%), piwi-interacting RNA (1.31%), transfer RNA (1.24%), small nuclear RNA (0.18%), and small nucleolar RNA (0.01%); fragments of coding sequence (1.36%), 5^′^ untranslated region (0.21%), and 3^′^ untranslated region (0.54%) were also present. In addition to the RNA composition of the libraries, we found that the three tested commercial kits generated a sufficient number of DNA fragments for sequencing but each had significant bias toward capturing specific RNAs.

**Conclusions:**

This study demonstrated that a wide variety of RNA species are embedded in the circulating vesicles. To our knowledge, this is the first report that applied deep sequencing to discover and characterize profiles of plasma-derived exosomal RNAs. Further characterization of these extracellular RNAs in diverse human populations will provide reference profiles and open new doors for the development of blood-based biomarkers for human diseases.

## Background

Many cells produce exosomes [1-3], small (30–100 nm) membrane vesicles that are released into the extracellular environment by fusing with the plasma membrane [4]. Although previously considered to be cellular waste products, emerging evidence indicates that exosomes can mediate diverse biological functions including angiogenesis, cell proliferation, tumor cell invasion and metastasis, immune response, and antigen presentation by the transfer of proteins, mRNAs and non-coding RNAs to neighboring or distant cells [3,5].

The existence of exosomes has been known for many years; however, it is only recently that these lipid-rich vesicles have been reported to contain an abundance of nucleic acids, in particular small non-coding RNAs [6]. Studies have now shown that the packaging of RNAs into exosomes is selective because the RNA profiles in exosomes do not fully reflect the RNA profiles observed in the parental cells [6-10]. When released from their cells of origin, exosomes may enter blood or other bodily fluids. To date, the microvesicles have been detected in blood (plasma and serum), bronchoalveolar lavage, urine, bile, ascites, breast milk, and cerebrospinal fluid [8,11-26]. These circulating vesicles can be taken up by recipient cells, allowing for cell-cell communication regardless of the distance between the cells. Exosome-mediated RNA transfer is believed to be an effective method for cell signaling and the exosomal RNA will certainly impact biological processes in the recipient cells [8,27-29].

Exosomal RNAs have been implicated in many exosome-mediated biological functions [30]. For example, RNAs delivered by exosomes prepared from X-ray treated cells were implicated in disseminating a bystander effect to target cells [31]. MicroRNAs (miRNAs) transferred by tumor-derived exosomes were reported to down-regulate the TAK1 pathway in hepatocarcinogenesis [32] and were pivotal in promoting tumor metastasis via a proinflammatory cytokine-driven expansion of myeloid-derived suppressor cells [33]. The let-7 miRNA family was selectively packaged into exosomes from a metastatic gastric cancer cell line and may have a role in the delivery of oncogenic signals to promote metastasis [34]. Exosomes derived from human (HMC-1) and mouse (MC/9) mast cell lines transported RNA to neighboring mast cells, impacting the function of the recipient mast cells [6,35]. The miRNAs transferred by the immune synapse were found to alter gene expression in the recipient antigen presenting cells [8]. These findings support the existence of a novel exosome-mediated mechanism by which one cell can regulate the activity or differentiation of other cells.

While exosomes have been shown to play functional roles in recipient cells, the RNA content of the exosomes may provide unique molecular signatures for disease diagnosis and prognosis [9,36-39]. It has been reported that exosomes from diseased individuals contained RNAs that were not found in healthy subjects [7,9]. These exosomes may carry RNA signatures that are characteristic of the parental cells, for example, tumor cells. So far, tumor-derived exosomes have been identified in the plasma of patients with lung adenocarcinoma, glioblastoma multiforme, malignant glioma, prostate cancer, and ovarian carcinoma ascites [9,10,30,40,41]. The association of exosomal miR-141 and miR-375 with metastatic prostate cancer has been confirmed in a cohort of patients with recurrent or non-recurrent cancer following radical prostatectomy [42]. These results suggest that circulating exosomes may provide a powerful tool for the non-invasive diagnosis and prognosis of human diseases.

Most of the current studies have used microarray or real time quantitative PCR (qPCR) assays to examine exosomal RNAs, with a focus on miRNAs. Because of the inherent limitations of these technologies, unknown miRNAs or other RNA species are often undetectable. Importantly, no systemic analysis of exosomal RNAs in peripheral blood has been reported until now. Blood is an important medium that allows exosomes to circulate and deliver cell signaling molecules to any part of the body. In this study, we performed a sequencing-based RNA profiling analysis using the blood from three blood donors. We evaluated three small RNA library preparation protocols and systemically characterized the extracellular RNA species. This study will provide a general guideline for blood-based exosomal RNA sequencing analysis and contribute to an understanding of exosome-mediated biological functions and mechanisms.

## Results

### Exosome size and RNA stability

**Figure 1:**
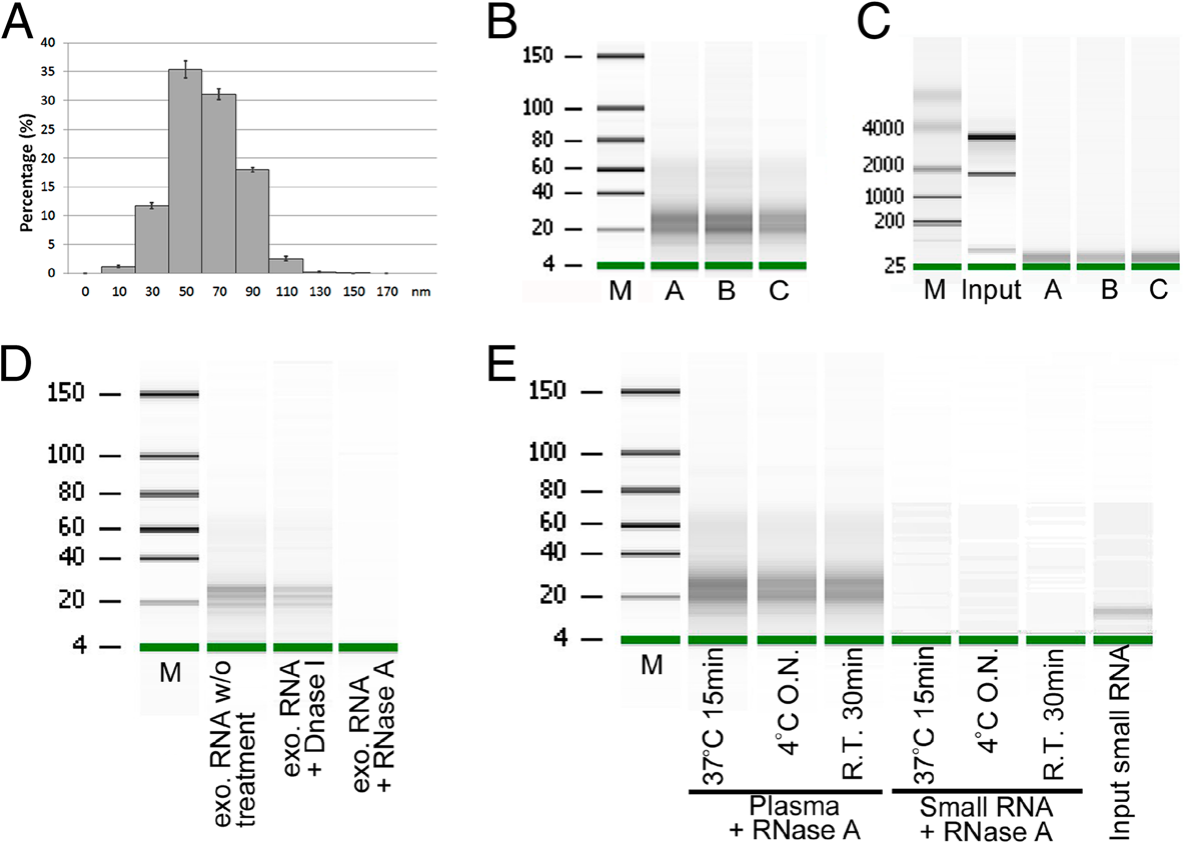
**Exosome isolation and exosomal small RNA quantification.** (**A**) Representative histogram of exosome size distribution. (**B**) Exosomal RNAs determined using the Agilent Small RNA Chip. The small RNAs were dominant in the exosomal RNAs. (**C**) Exosomal RNAs determined by the Agilent RNA Pico Chip. Exosomal RNA samples **A**, **B** and **C** contained no detectable 18S and 28S rRNAs. Cellular RNA from HEK293 was loaded as positive control for 18S and 28S rRNAs (**D**) Isolated exosomal RNAs treated either with DNase I or RNase A. (**E**) Plasma and control small RNAs treated under various conditions.

We used the NanoSight LM10 instrument to determine the size distribution and concentration of the exosomes. For the three samples tested, the exosome sizes ranged from 30–90 nm (Figure 1A). The number of exosomes per 250 μL of plasma ranged from 0.21–1.08 × 10^8^ and the RNA yields from each of the samples were similar, ranging from 10–15 ng (as determined using an Agilent Bioanalyzer). The RNAs sizes ranged from 18–28 nucleotides (nt) (Figure 1B). We repeated the RNA extraction at least twice for each sample. The RNA size distributions and yields were consistent both between extractions and between samples. We also ran an Agilent RNA 6000 Pico chip and found no evidence of cellular RNA contamination (Figure 1C). In subsequent enzyme protection assays, we treated the isolated nucleic acids with DNase I and found that there was no significant degradation; however, when treated with RNase A, the isolated nucleic acids were completely degraded (Figure 1D). To test whether or not the exosome membrane protected RNA from RNase A degradation, we treated plasma samples with RNase A under various conditions and obtained high yield of RNAs in the samples after the treatment (Figure 1E).

### Comparison of three small RNA library preparation protocols

**Figure 2:**
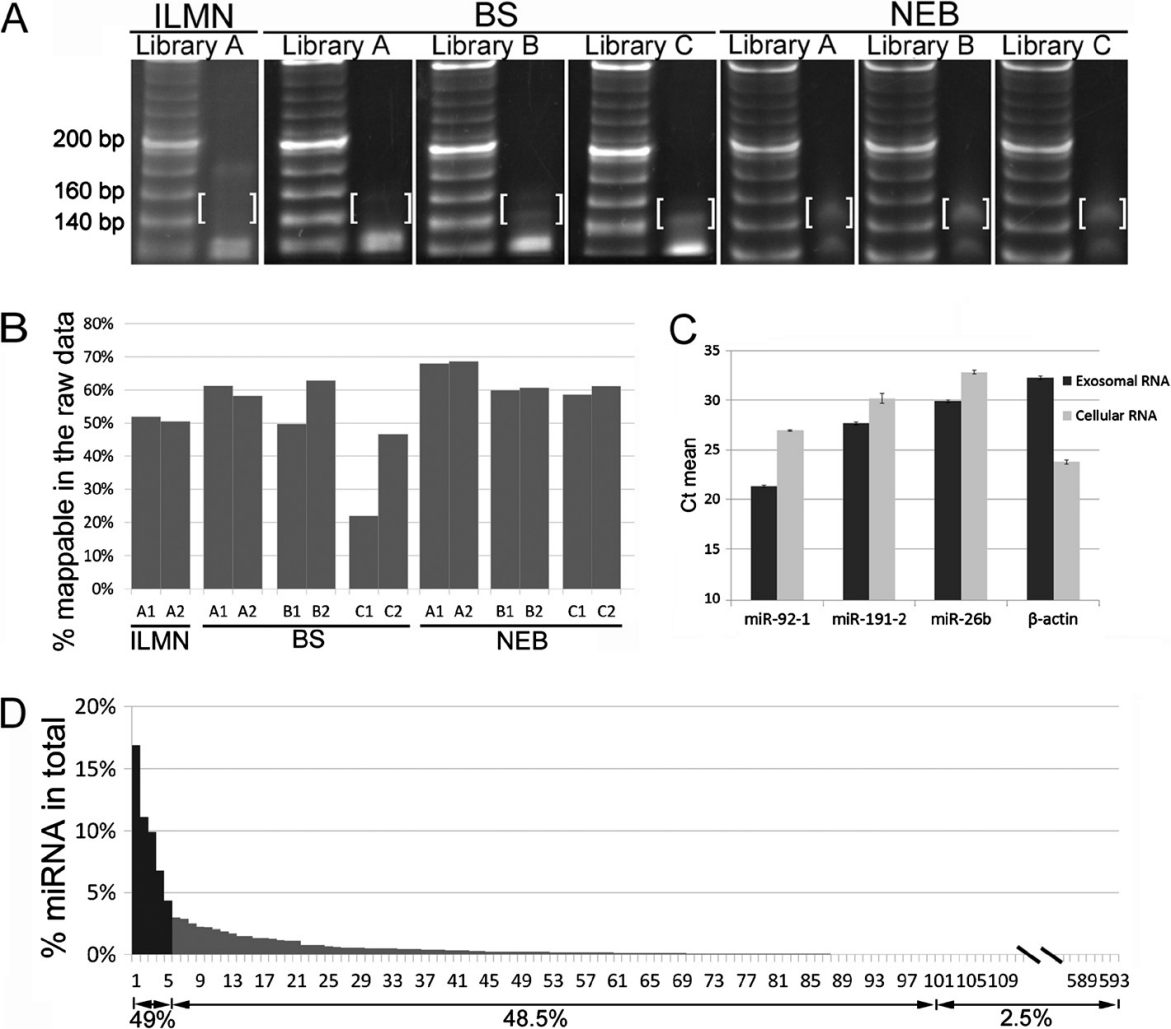
**Library preparation and analysis of the raw sequencing data.** (**A**) PAGE analysis of the sequencing libraries prepared using three different kits. 20-bp DNA ladders are shown on the left of each panel while the prepared libraries are shown on the right. The anticipated RNA sequencing constructs ranging from 140–160 bp are highlighted within brackets. The DNA bands (~125 bp) are the adaptor dimers. (**B**) Percentage of mappable RNAs in the raw sequencing reads. (**C**) Abundance of the selected miRNAs and β-actin in plasma exosomal RNA and HEK293 cellular RNA. Gene and miRNA abundance was determined by the threshold cycle (Ct), where Ct values >30 are defined as rare. (**D**) Percentage of each miRNA in the repertoire of total miRNA reads.

To compare three commercially available library preparation kits, we constructed sequencing libraries using 2 ng of exosomal RNA and 15 PCR cycles for all the preparations. We found that there were significant differences in the size distribution of the amplified libraries when comparing the three different preparation protocols. Each of the protocols was expected to have sequencing library size of 140–160 bp. Among these kits, the NEBNext multiplex small RNA library preparation kit (NEB) produced more target fragments that were separated from adaptor dimers (Figure 2A). The Illumina kit constantly generated a strong DNA fragment of ~180 bp, but the target fragments were hardly seen. The Bioo Scientific kit generated fragments of the expected size, but separation with adaptor dimers was poor. Although all three kits generated enough DNA at the targeted size for sequencing, the pre-sequencing qPCR results showed that the NEB kit produced the highest yield of recovered RNA-seq libraries with less variation.

### Data processing and genome mapping

We replicated each of the three samples and tested each replicate in at least two separate library preparation protocols. In all we produced a total of 14 sequencing libraries (7 replicates). From the 14 libraries, we obtained a total of 101,804,712 raw single-end reads, an average of about 7.27 million reads per library. Among the raw reads, we found 73,112,422 (71.82%) sequences with insert lengths ≥16nt (query sequences) and of these, 55.54% (56,542,143) could be mapped to known RNAs and to the human genome (Table 1). In 12 of the 14 libraries, at least 50% of the raw reads were mappable sequences (Figure 2B). On average, the NEB libraries had the highest percentage of mappable reads (62.72%) while the Bioo Scientific and Illimina libraries had 50.07% and 51.15% mappable reads, respectively. The size distribution of the inserts also varied among these kits. The NEB libraries had the highest proportion of 21–23 nt inserts, followed by Illumina and Bioo Scientific kit [see Additional file 1]. Nevertheless, in all three libraries, miRNA sequences were the most common, accounting for 76.20% of the mappable reads (Table 1).

**Table 1:** Sequence read counts from RNA sequencing for the 14 libraries

**Kit**	**Samples**	**Raw reads**	**Query reads**	**Mappable reads**	
				**miRNAs (%)**	**Others (%)**
Illumina					
	A1	5,038,311	3,629,688	2,003,390(76.7)	607,629(23.3)
	A2	5,643,622	3,753,814	2,030,007(71.3)	819,077(28.7)
Bioo Scientific					
	A1	6,506,144	4,850,351	3,395,109(85.3)	587,039(14.7)
	A2	8,143,961	5,632,208	3,998,520(84.4)	741,200(15.6)
	B1	7,586,416	5,129,990	2,847,137(75.4)	927,909(24.6)
	B2	6,365,053	5,246,494	3,111,194(77.8)	885,208(22.2)
	C1	9,039,025	2,446,141	1,490,184(75.3)	487,517(24.7)
	C2	5,622,703	3,270,113	2,006,209(76.6)	612,942(23.4)
NEB					
	A1	7,990,894	6,575,021	4,419,868(81.5)	1,004,342(18.5)
	A2	8,038,976	6,605,662	4,507,678(81.8)	1,000,301(18.2)
	B1	7,257,368	6,342,279	2,653,105(61.2)	1,684,951(38.8)
	B2	8,268,147	7,302,007	3,107,916(62.1)	1,898,996(37.9)
	C1	9,429,406	7,138,699	4,219,991(76.5)	1,298,809(23.5)
	C2	6,874,686	5,189,955	3,294,935(78.5)	900,978(21.5)
Total		101,804,712	73,112,422	43,085,243(76.2)	13,456,898(23.8)

### Exosomal miRNA content

Because the abundance of most miRNAs is low in the exosomes, we defined detectable miRNAs as those that had at least one sequence per million mappable miRNA reads. Accordingly, we detected a total of 593 known miRNAs in the 14 libraries. In each individual library, the number of detectable known miRNAs varied from 380 to 474 with an average of 419 [see Additional file 2]. To validate the sequencing data, we selected three miRNAs with different read counts for qPCR quantification; namely, miR-92a-3p, which had a high read count, and miR-191-3p and miR-26b-5p, which had relatively low counts. HEK293 cellular RNA was used as a positive control. The results showed that the expression levels of miR-92a-3p and miR-191-3p were 385 and 4.6 fold higher than the expression level of miR-26b-5p (Figure 2C). These relative abundance ratios were close to the ratios from the sequencing data using the Illumina kit (441 fold for miR-92a-3p versus miR-26b-5p, and 10.7 fold for miR-191-3p versus miR-26b-5p). As anticipated, the expression level of β-actin was extremely high in cells, but hardly detectable in the exosomes. The five most abundant miRNAs in the libraries were miR-99a-5p, miR-128, miR-124-3p, miR-22-3p, and miR-99b-5p, which together accounted for 48.99% of all detectable miRNAs (Table 2). The 100 most abundant miRNAs made up 97.47% of the detectable miRNA sequences; therefore, the remaining 493 low abundant miRNAs accounted for only 2.53% (Figure 2D).

**Table 2:** **The twenty most abundant miRNAs among the plasma exosomal RNAs** (**normalized read counts per million mappable miRNAs**)

**miRNA**	**ILMN**		**Bioo Scientific**						**NEB**					
	**A1**	**A2**	**A1**	**A2**	**B1**	**B2**	**C1**	**C2**	**A1**	**A2**	**B1**	**B2**	**C1**	**C2**
hsa-miR-99a-5p	17604	16094	211064	186965	259315	347052	185255	186911	123880	127903	178157	181267	148618	156684
hsa-miR-128	23296	24353	13735	5650	1634	8538	3164	3488	240820	246854	196217	198956	271367	289239
hsa-miR-124-3p	3965	4924	172880	202866	272353	142389	294247	227036	7409	6878	8012	8298	5548	5935
hsa-miR-22-3p	88942	90113	104977	114037	89815	74092	103413	105707	22263	23545	30113	31787	25136	27245
hsa-miR-99b-5p	147955	140925	19482	22631	70340	51141	30682	28513	12113	12021	16975	16029	14154	12804
hsa-miR-181a-5p	71811	74929	30810	27391	16161	18060	19530	22364	18741	20040	24994	25733	19369	21147
hsa-miR-9-3p	2452	2261	47411	45386	15754	21748	37897	51888	25442	24983	34070	34203	26904	26030
hsa-miR-100-5p	29843	26438	34978	13511	15176	71812	11906	13708	20410	20201	23359	22036	21868	19371
hsa-miR-129-5p	1239	1250	1499	2226	2018	1263	3468	3002	36690	38005	65007	65498	44690	43875
hsa-miR-125b-5p	16643	14233	41087	38871	28081	37343	13492	16907	18291	17186	13985	13735	16600	14989
hsa-miR-9-5p	1660	1722	4935	6023	3524	3777	9878	10890	55445	49993	33259	32108	35887	30142
hsa-miR-27b-3p	42215	44272	14583	18653	8155	8121	10445	12335	18378	18108	14894	14884	16922	16602
hsa-miR-486-5p	103037	110538	4378	3697	565	1518	1087	1538	2605	2452	575	559	704	675
hsa-miR-181b-5p	12367	12732	11171	10640	6771	8423	9149	11734	17762	18681	23870	24021	19829	20536
hsa-miR-125a-5p	64362	48420	11387	13447	7045	9135	5385	6101	10799	9895	2968	2867	8081	6412
hsa-miR-127-3p	29214	32718	14617	13023	15528	20062	7562	9800	7162	7135	7944	7654	7082	6660
hsa-miR-92b-3p	56679	56085	6098	8510	13143	7791	10936	9280	2522	2413	3184	3311	3030	2921
hsa-let-7i-5p	4741	5176	6153	6734	3550	3128	5004	5179	34309	31210	19278	18343	18410	15844
hsa-miR-320a	1525	1553	6928	6594	7967	10042	6811	9426	14608	14321	26781	25923	17449	14913
hsa-let-7b-5p	1354	1395	4754	4563	3452	4111	1845	2568	21801	25102	22716	22410	21734	23257

### Variability of miRNAs between technical replicates, samples, and preparation protocols

**Figure 3:**
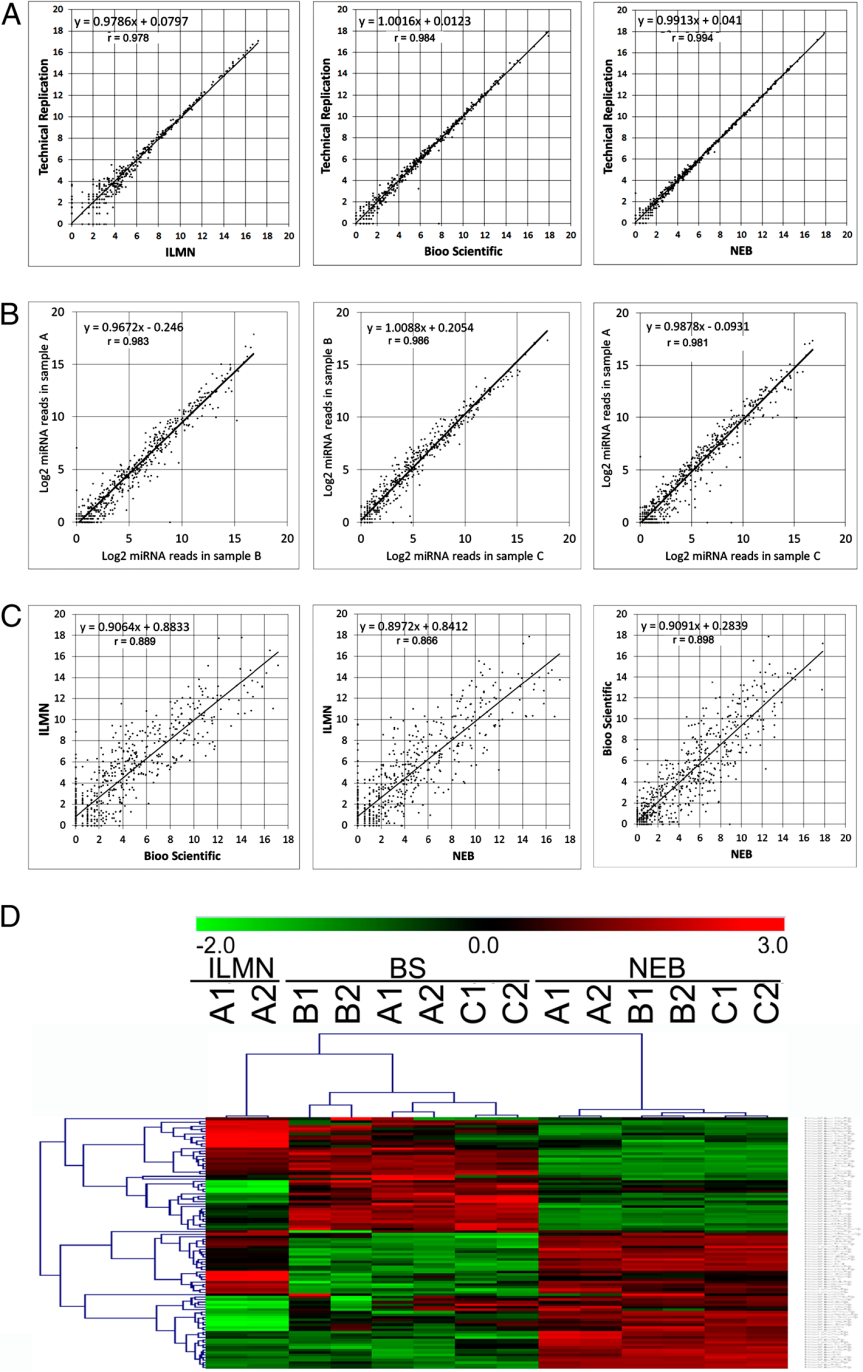
**miRNA correlations between technical replication, biological replication and methodological replication.** (**A**) Scatter plots of technical replicates in sample A. Illumina kit (ILMN), left panel (r = 0.978); Bioo Scientific kit (BS), middle panel (r = 0.984); and NEB kit, right panel (r = 0.994). (**B**) Scatter plots of sample **A** and sample **B** (left panel, r = 0.983); sample **B** and **C** (middle panel, r = 0.986), and sample **A** and **C** (right panel, r = 0.981). Only the plots for the pooled samples are shown. (**C**) Scatter plots of ILMN and BS (left panel, r = 0.889), ILMN and NEB (middle panel, r = 0.866), and BS and NEB (right panel, r = 0.898). Only the plots for the pooled samples are shown. (**D**) Heat map of unsupervised hierarchical clustering of the 100 most abundant miRNAs. Samples and library preparation methods are indicated on horizontal axis at the top of the heat map. miRNAs are indicated vertically on the right.

To examine the variations of the miRNA contents that may be produced by potential technical and biological variability, we performed a correlation coefficient analysis using log2 transformed values after normalizing reads to per million counts. For the seven pairs of technical replicates, the overall reproducibility was excellent regardless of the kits used (average Pearson correlation coefficient r = 0.98) (Figure 3A). The highest correlations were observed between the NEB replicates (r = 0.994), followed by the Bioo Scientific (r = 0.984) and Illumina (r = 0.978) replicates.

For sample-sample (biological) variations, we compared pooled samples (samples A, B and C) prepared using the Bioo Scientific and NEB kits. Overall, we found that there was significant correlation between samples B and C (r = 0.986) (Figure 3B), followed by between samples A and B (r = 0.983), and then between samples A and C (r = 0.981). However, when comparing the variations among the different library preparation protocols, we found striking differences although the average correlation coefficient r value was close to 0.884. The correlation r values were 0.898 between NEB and Bioo Scientific (Figure 3C), 0.889 between Bioo Scientific and Illumima, and 0.866 between NEB and Illumima.

To better demonstrate the technical, biological, and methodological variations, we performed an unsupervised hierarchical clustering analysis using the log2-transformed sequence counts of the 100 most abundant miRNA transcripts. As expected, the heat map showed that there was a clear separation between groups composed of replicates, samples and library preparation kits (Figure 3D). Nearly all of the 100 miRNAs showed similar expression patterns between technical replicates; however, some of them showed significant variations among different samples and most showed differences among different preparation kits. For example, the NEB kit detected over 21 fold more miR-129-5p sequences than either the Illumina or the Bioo Scientific kits. The Illumina kit generated over 50 fold more miR-486-5p sequences in sample A than either the Bioo Scientific or the NEB kits. The Bioo Scientific kit produced over 31 fold more miR-124-3p sequences than either the Illumina or the NEB kits. The methodological variations were also evident for the top 20 most abundant miRNAs (Table 2).

### Sharing of detectable miRNAs

**Figure 4:**
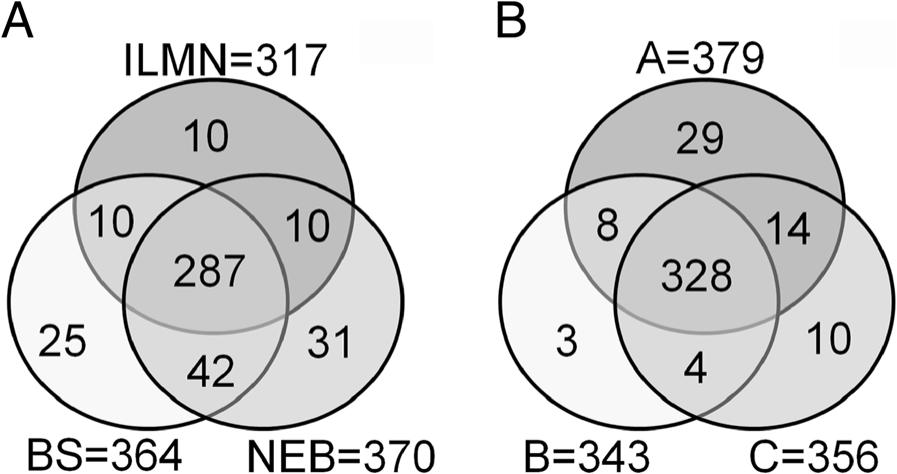
**Venn diagrams showing miRNAs that are common in the three samples.** (**A**) Unique and shared miRNAs in sample **A** in the libraries prepared using the different kits. (**B**) Unique and shared miRNAs in the different samples. The miRNAs with read counts ≥5 per million mappable sequences were used for the comparison.

To examine if the miRNAs were unique to or common to the different preparation protocols, we first removed the low abundant miRNAs (<5 normalized counts per million miRNA reads) and then compared the remaining miRNAs among the three kits. Sample A was used for the comparison because this was the only sample that was tested in all three kits. The Illumina, Bioo Scientific and NEB kits detected 317, 364 and 370 known miRNAs, respectively. Of these, 287 were detected by all three kits (Figure 4A). In addition to the miRNAs that were shared, we also identified some miRNAs that were unique to one particular library preparation protocol. For example, miR-2964-3p and miR-3065-5p were detected only by the Bioo Scientific kit. However, the abundance of the unique miRNAs were generally low in the libraries [see Additional file 2].

Additionally, we examined different samples for miRNA that were shared. Samples A, B and C each had 379, 343 and 356 miRNAs with >5 reads per million, respectively and 328 of them were shared among the three samples. Samples A, B and C also had 29, 3, and 10 unique miRNAs, respectively (Figure 4B). However, similar to the findings for the methodological differences, most of the sample-specific miRNAs were low in abundance.

### Other RNA species

**Figure 5:**
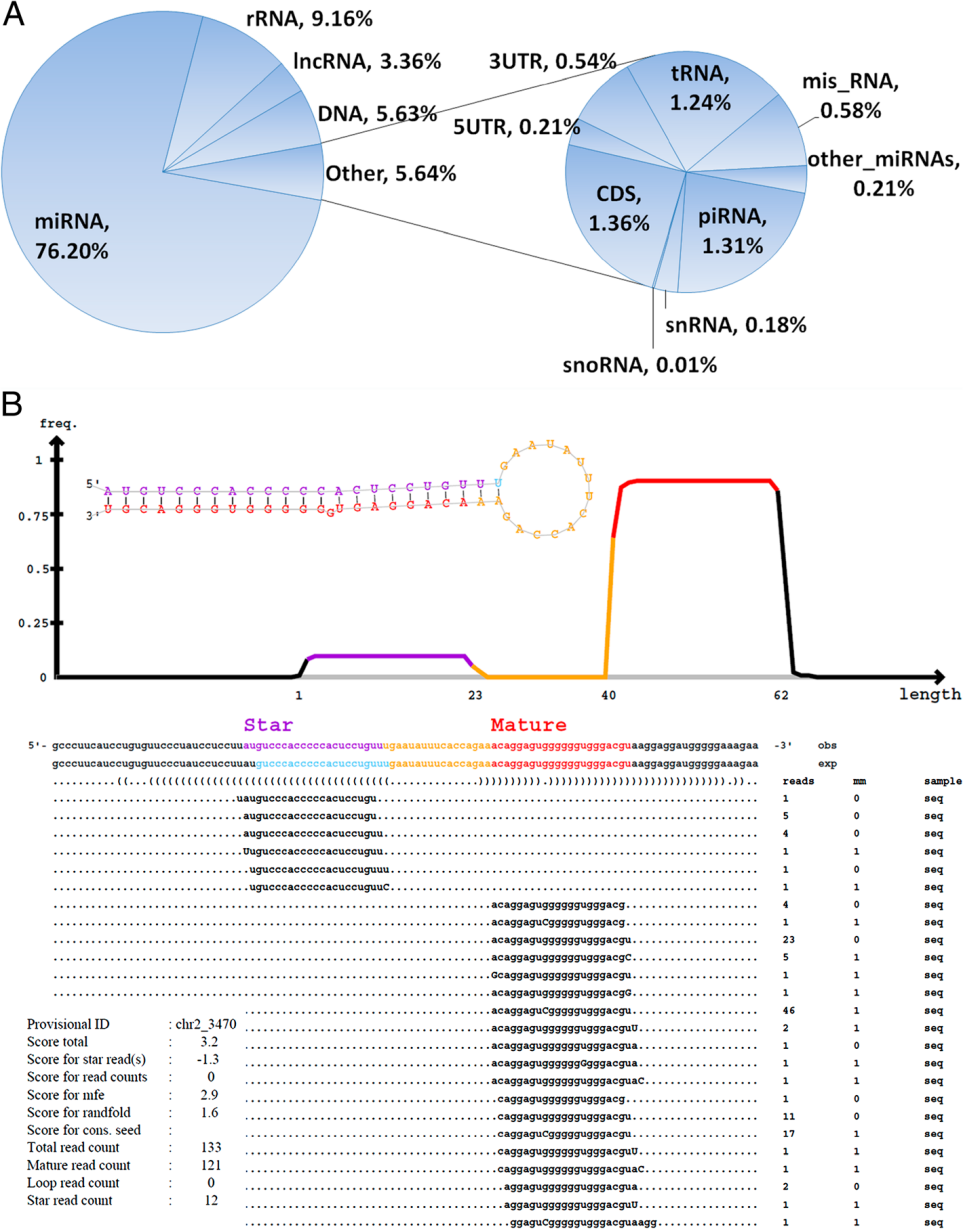
**Other RNA species that were detected in the exosomal RNA libraries.** (**A**) Pie chart of RNA species and their distributions in the plasma-derived exosomes. Misc RNAs are the RNA sequences that mapped to the human genome but not in any of the categories listed. The DNA category represents the novel transcripts that have no annotation in the human RNA database. (**B**) Graphic and statistics of a representative novel miRNA predicted by miRDeep2. Both star and mature strands were detected and integrated. Lower left table shows information about the sample and the miRDeep2 scores, along with the read count for each component of the putative miRNA. mm, number of mismatches. Mismatched nucleotides are indicated by uppercase letters.

To annotate the exosomal RNA species that were not identified as miRNA transcripts, we first removed all the known miRNA sequences from the libraries and then mapped the remaining sequences to the human genome that had RNA annotations. Figure 5A shows the percentage of other small non-coding RNAs, tRNA, rRNA, small nuclear (snRNA), small nucleolar (snoRNA) and piwi-interacting RNA (piRNA) that were detected. The rRNA was the most common among them, accounting for 9.16% of all mappable counts, followed by piRNA (1.31%), tRNA (1.24%), snRNA (0.18%), and snoRNA (0.01%). Clearly, the exosomes contained relatively low levels of rRNA, which is in contrast to a typical eukaryotic cell where rRNA makes up at least 80% of the total RNA molecules. Interestingly, we also detected low levels of “long” RNA in the small RNA libraries. We detected 3.36% of long non-coding RNA (lncRNA), 1.36% of coding sequences (CDS), 0.54% of 3′untranslated region (UTR) and 0.21% of 5′UTR sequences [see Additional file 3]. Compared to cellular RNA components, the CDSs in the exosomal RNAs accounted for a much smaller fraction of all mappable sequences. In addition, we found 0.21% of other RNA sequences that mapped to species other than human. For instance, the bovine bta-miR-6529 was the most common non-human miRNA found in the 14 libraries. However, this result should be interpreted with caution because most, if not all, of “non-human” RNAs may be an artifact [43].

The most abundant of the gene fragments that contained the CDS, 5′UTR or 3′UTR sequences that were found in the small RNA libraries were involved in fundamental metabolic processes [see Additional file 3]. For example, the most common CDS sequences mapped to *NYNRIN* [GenBank:NM_025081] and *LARS2* [GenBank:NM_015340], both of which encode proteins that participate in tRNA or rRNA metabolism. The most frequent 5′UTR sequence mapped to *PVRL2* [GenBank:NM_001042724], which encodes a protein that is involved in the cell to cell spreading of herpes simplex virus and pseudorabies viruses [44]. The second most common 5′UTR sequence mapped to *ENTPD4* [GenBank: NM_004901], which encodes an endo-apyrase that is capable of cleaving nucleoside tri- and/or di-phosphates [45]. The most frequent 3′UTR sequence mapped to *PAQR5* [GenBank:NM_017705], which encodes progestin and adipoQ receptor family member V, which functions as a membrane progesterone receptor [46].

### Predicted novel miRNAs

To identify novel miRNAs in the 14 libraries, all the raw data were processed independently using miRDeep2 [47]. The miRDeep2 software detected 185 distinct novel miRNAs in the 14 libraries and 15, 88 and 111 novel miRNAs in the individual libraries generated by the Illumina, Bioo Scientific and NEB kits, respectively [see Additional file 4]. Among the putative miRNAs, two were common to libraries prepared with the Illumina and NEB kits, six were common to the Illumina and Bioo Scientific libraries, and 22 were common to the NEB and Bioo Scientific libraries. Of the 15 putative miRNAs in the Illumina libraries, four (26.7%) were found in technical replicates. For the Bioo Scientific-derived library, 16 of the 88 novel miRNAs (18.2%) were found in at least two replications and 19 (21.6%) were common to at least two samples. For the NEB-derived libraries, 33 of the 111 novel miRNAs (29.7%) were present in technical replicates and 38 (34.2%) were common to at least two samples. A representative readout of the predicted miRNAs from an NEB library is shown in Figure 5B. Multiple reads of both the mature and star miRNA sequences (typical components of a miRNA) were found in this library. All of the predicted miRNAs had the typical miRNA features at genomic DNA level.

### Potential regulatory roles of exosomal miRNAs

We performed gene enrichment analysis using a set of genes that were predicted to be targets of the highly abundant miRNAs in the exosomes. The miRDA tool (http://mirdb.org/) predicted a total of 1205 target genes for the top five exosomal miRNAs. We found significant enrichment of these genes in gene ontology (GO) terms, including protein phosphorylation, RNA splicing, chromosomal abnormality, and angiogenesis. For example, we found a 1.33 fold enrichment of phosphoproteins (Bonferroni p = 1.35E-17), a 1.23 fold enrichment of splice variants (Bonferroni p = 3.43E-7), and a 2.46 fold enrichment of genes involving chromosomal rearrangement (Bonferroni p = 5.81E-5) (Table 3). Interestingly, we also observed significant enrichment in vasculature development (2.22 fold enrichment, Bonferroni p = 3.75E-2) and neurotrophin signaling pathway (2.68 fold enrichment, Boferroni p = 8.07E-3).

**Table 3:** miRNA target enrichment analysis

**Term**	**Fold enrichment**	**p value**	**Bonferroni p value**
phosphoprotein	1.33	2.51E-20	1.35E-17
splice variant	1.23	1.20E-10	3.43E-07
chromosomal rearrangement	2.46	1.08E-07	5.81E-05
nucleotide phosphate-binding region: ATP	1.68	2.38E-07	6.80E-04
mutagenesis site	1.40	1.51E-06	4.31E-03
kinase	1.76	1.82E-06	9.83E-04
cytoplasm	1.27	9.43E-06	5.08E-03
vasculature development	2.22	1.26E-05	3.75E-02
neurotrophin signaling pathway	2.68	4.94E-05	8.07E-03
endosome	2.01	5.44E-05	2.44E-02

## Discussion

Exosomes circulating in the blood carry regulatory RNA molecules, thereby allowing for long distance cell-cell communication. Because diseased cells, including tumor cells, actively release exosomes into the blood stream, the circulating exosomes may provide a stable source of RNAs for disease diagnosis, prognosis and treatment management [2,6,39,48]. In this study, we developed a protocol for isolating exosomal small RNA from a very low volume of plasma. We performed deep sequencing analysis of the exosomal RNAs, and generated expression profiles of the important extracellular RNAs. Our findings will not only help characterize the RNA content of exosomes but will also contribute to understanding exosome function and biology.

Exosomal RNA profiling analysis is not possible without high quality RNA. Compared to cellular RNAs, exosomal RNAs are more stable [49], and are reportedly resistant to physical degradation such as prolonged storage and freeze/thaw cycles [50]. The circulating exosomal RNAs have been found to be resistant to biochemical degradation by ribonuclease in serum as well as by RNase A under an in vitro condition. This stability makes reproducible and consistent evaluation of blood-based non-coding RNA possible [51]. Indeed, our study strongly supports the protective role of the microvesicles or other proteins in the stability of the circulating plasma RNA. Recently, Argonaute 2 was reported to bind and protect miRNAs from degradation in the circulation [52]. It appears that Argonaute 2-protected miRNAs contribute to a significant proportion of the RNA circulating in the blood. Therefore, RNAs (at least miRNAs) in the blood stream are protected by multiple mechanisms and may be more stable than previously believed [53].

The dominant size of the exosomal RNA that was detected in this study was 18–28 nt. This size range is apparently smaller than that of the small RNAs derived from culture medium [34,54,55], where the sizes were centered at about 70 nt. Different isolation methods may account for the size discrepancies. Ultracentrifugation at 100,000 g seems to be less capable of discriminating exosomes from other microvesicles, especially when the exosomes are large. The mixed sizes of the isolated microvesicles may have caused more heterogeneity of RNA biotypes, which in turn impacted on the size and abundance of the RNAs in the libraries. In addition, the ExoQuick-based assay that we used to precipitate the exosomes may co-precipitate non-exosomal microparticles or RNA-binding proteins. Therefore, technically, the exosomal RNA may account for a fraction of all RNAs isolated by this assay. To obtain reproducible and reliable expression data, further study of the isolation methods is highly recommended.

The highly enriched exosomal miRNAs may have significant impacts on the target cells. For example, miR-99a-5p, the most abundant miRNA in the plasma exosomes, functions in a tissue-dependent manner. In prostate tumor tissue, miR-99a-5p was found to be down-regulated and its overexpression in a prostate cancer cell line was reported to inhibit the growth of the recipient cells and decreased the expression of the prostate-specific antigen [56]. However, overexpression of the miR-99a was also reported to be responsible for increased proliferation, migration and fibronectin levels in a murine epithelial cell line NMUMG, possibly via modulating the TGF-β pathway [57]. The functional role of miR-124 as a tumor suppressor has been established in glioblastoma, breast cancer, hepatocellular carcinoma, gastric cancer, and prostate cancer [58-62]. Another study demonstrated that miR-124 silencing in neuroblastoma cells led to cell differentiation, cell cycle arrest and apoptosis [63]. In support of the important functions of the highly expressed exosomal miRNAs, our GO-based target prediction showed their potential roles in phosphorylation, RNA splicing, chromosomal abnormality, and angiogenesis; however, these predictions need further functional confirmation. Clearly, once released into target cells, the highly enriched miRNAs may participate directly in the regulation of mRNA translation and influence cell functions.

We also observed low level of “long” RNA fragments such as mRNA and lncRNA in the small RNA sequencing libraries. Our library preparation protocols were designed to capture small non-coding RNAs (~20–40 nt long). Therefore, the mRNAs and lncRNAs that were identified in this study should all be treated as fragmented RNAs. The procedures that were used for RNA extraction and library preparation may have caused partial RNA degradation, enabling the detection of fragments of the long RNAs in the small RNA libraries. Another possible explanation for the presence of long RNA fragments is that the exosomes also function as a “reservoir” to remove degraded mRNA and lncRNA derived from the cytosol. The exact mechanism underlying the presence of fragmented long RNAs in exosomes remains to be unraveled.

The current study demonstrated the reproducibility for each library preparation kit. Both Pearson correlation and hierarchical cluster analysis showed highly correlated RNA profiles between technical replicates, suggesting the consistency of these commercial kits. However, the study also showed significant biases between the library preparation methods. Each kit preferentially captured specific RNA sequences. For high abundant RNAs, this bias does not seem to be problematic because all three kits detected these RNAs. For low abundant RNAs, however, the bias could be an issue because these RNAs may be detected by one kit but not by another. Protocol-based bias may also create problems in data interpretation if different commercial kits are used. We suggest that separate validation using qPCR should be performed for all sequencing-based detections.

The ever growing number of novel sequences in the miRNA database implies that human miRNA annotation is far from complete [64]. To identify novel miRNAs, next generation sequencing is the most powerful and the most popular approach. However, systematic bias during library preparation and the limited power of prediction algorithms means that some of the novel miRNAs may have been falsely predicted. We strongly recommended using other complementary methods such as Northern blot and qPCR for subsequent validation. Additionally, this study used only three plasma samples and, therefore, our findings may not fully represent all exosomal RNAs in human populations. To completely survey the exosomal transcriptome more samples from diverse populations and with different disease status are required.

The plasma exosomes are believed to be derived from a variety of cell populations. Their heterogeneous origin may limit the detection of disease-specific exosomes in peripheral blood samples. Vast numbers of exosomes shed from other cell types may dilute the exosome population derived from tumor cells, significantly reducing the proportion of tumor-derived miRNAs in the sequencing libraries. Because the less common tumor-derived miRNA may be a direct reflection of the disease status and critical for tumor development, the increased read depth of RNA sequencing is required. It is worth mentioning, that although the detection of rare RNA transcripts will increase as sequencing depth increases, the rare sequences still account for a tiny fraction of the exosomal RNAs. Whether or not the rare exosomal miRNAs are functional remains to be determined.

## Conclusions

We developed a comprehensive data-generation and data-analysis pipeline that includes exosome isolation, RNA extraction, library preparation, RNA sequencing, and RNA annotation. Our data show that plasma-derived exosomes contain diverse RNA species, in particular, miRNA. The abundance of the exosomal RNAs varies dramatically. Some highly abundant miRNAs may play critical roles after being transferred to target cells. The three commercial small RNA preparation kits that we tested generated sufficient DNA fragments for sequencing but had significant biases towards capturing specific RNAs. The use of large-scale RNA sequencing will ensure the discovery and characterization of the whole transcriptome (known and unknown RNAs) of the blood-derived exosomes, which has not been completely examined so far. A fully characterized transcriptome will help gain a better understanding of exosome-mediated molecular mechanisms and will contribute to biomarker discovery. It is expected that the blood-based sequencing assay described here will find clinical applications as a biomarker discovery tool for disease diagnosis and prognosis.

## Methods

### Study design and participant consent

**Figure 6:**
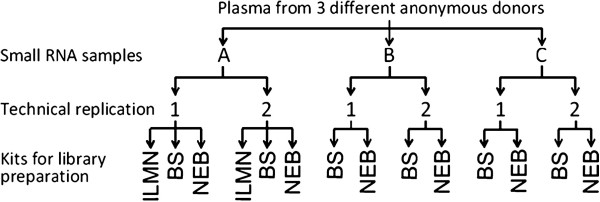
**Schematic illustration of the study design.** Three plasma samples (**A**, **B** and **C**) were used in this study. Each sample had a technical replicate. Three commercial kits were tested: ILMN-Illumina, BS-Bioo Scientific and NEB-New England Biolab.

The objective of this study was to provide general guidelines for profiling analysis of exosomal RNA in peripheral blood. To accomplish this goal, we selected plasma samples (samples A, B and C) from three anonymous blood donors and split each sample into two for technical replication. We tested the six samples (A1 and A2, B1 and B2, C1 and C2) using two small RNA library preparation kits: NEBNext multiplex small RNA library preparation kit (NEB, New England Biolab, Ipswich, MA, USA) and NEXTflex small RNA sequencing kit (Bioo Scientific, Austin, TX, USA). We also tested samples A1 and A2 using the TruSeq small RNA sample preparation kit (Illumina, San Diego, CA, USA). Altogether, we tested the six plasma samples by sequencing the 14 indexed libraries prepared using the three kits as described above. This study allowed the direct comparison of three currently available small RNA library preparation protocols and identified the most suitable strategy for future exosomal RNA sequencing analysis. A flowchart of the study design is shown in Figure 6. The participants gave written informed consent for their blood to be used for this study. The use of the human biospecimens was approved by the Institutional Review Board of the Medical College of Wisconsin and the Mayo Clinic.

### Exosome isolation

Human plasma samples were obtained from the Mayo Clinic and stored at -80°C before use. Exosomes were isolated from 250 μL of plasma using the ExoQuick exosome precipitation solution (System Biosciences, Mountain View, CA, USA) according to the manufacturer’s instructions with minor modifications. Briefly, the plasma was incubated with thromboplastin D (Thermo Scientific, Middletown, VA, USA) for 15 min at 37°C. After centrifugation at 10,000 rpm for 5 min, the supernatant was mixed with 75 μL of ExoQuick solution and RNase A (Sigma, St. Louis, MO, USA) to a final concentration of 10 μg/mL. The mixture was kept at 4°C overnight and then further mixed with 150 units/mL of murine RNase inhibitor (NEB) before centrifugation at 1500 g for 30 min. The exosome pellet was dissolved in 25 μL 1 × PBS; 2 μL of the solution was reserved for evaluation of exosome size and concentration using the NanoSight LM10 instrument (Particle Characterization Laboratories, Novato, CA, USA), and RNA was extracted immediately from the remaining solution.

### Exosome quantitation and size determination

The concentration and size distribution of the isolated exosomes were measured using NanoSight. Prior to sampling, the sample solutions were homogenized by vortexing, followed by serial dilution to a final dilution of 1:100,000 in 0.2 μm-filtered 1x PBS. The National Institute of Standards and Technology (NIST) traceable 97 nm ± 3 nm polystyrene latex standards were added and analyzed along with the diluted exosome solution to validate the operation of the instrumentation. A blank 0.2 μm-filtered 1x PBS was also run as a negative control. Each sample analysis was conducted for 90 seconds. The Nanosight automatic analysis settings (high sensitivity, blue laser [405 nm, 645 mW]) were used to process the data. All samples were evaluated in triplicate.

### RNA isolation

Exosomal or HEK293 cellular RNA was prepared using a miRNeasy Micro Kit (QIAGEN, Valencia, CA, USA). Twenty-three μL of exosome suspension or 1 × 10^6^ HEK293 cells were mixed with 700 μL QIAzol lysis buffer, and the mixture was processed according to the manufacturer’s standard protocol. The extracted RNA was eluted with 14 μL of RNase-free water. The quantity and quality of the RNA were determined by Agilent Bioanalyzer 2100 with a Small RNA Chip for exosomal RNA, and a RNA 6000 Pico Kit for cellular RNA (Agilent Technologies, Santa Clara, CA, USA).

### Enzyme protection assay

RNA isolated from the plasma exosomes was first incubated at room temperature, either with 30 units/μL of DNase I (QIAGEN) for 10 min or with 10 μg/mL RNase A for 30 min. The RNase A digestion was terminated by adding 150 units/mL of murine RNase inhibitor. The resultant RNA samples were processed with the Agilent Bioanalyzer. In another enzyme protection assay, before the addition of murine RNase inhibitor, plasma samples were incubated with 10 μg/mL RNase A under various conditions, namely, at 37°C for 15 min, at room temperature for 30 min, or at 4°C overnight, followed by exosome isolation and RNA extraction. The same procedure was carried out using commercially available small RNA, which acted as a control for this assay. The RNA eluents along with the naked small RNase A-treated RNA were then evaluated with the Agilent Bioanalyzer.

### RNA library preparation

For each library, 2 ng of small RNA was used in all the experimental procedures. Each library was prepared with a unique indexed primer so that the libraries could all be pooled into one sequencing lane. The 14 RNA libraries were prepared and amplified following the instruction of each manufacturer. The amplified libraries were resolved on a native 5% acrylamide gel. DNA fragments from 140–160 bp (the length of miRNA inserts plus the 3′ and 5′ adaptors) were recovered in 12 μL elution buffer (QIAGEN). The indexed libraries were quantified on the Bio-Rad 1000 qPCR instrument using the KAPA Library Quantification Kit in triplicates, according to the manufacture’s protocol (Kapa Biosystems, Woburn, MA, USA). Ten μL of the pooled library at a final concentration of 2 nM were then sent to the Core Facility at Medical College of Wisconsin for sequencing using Illumina HiSeq2000 DNA sequence analyzer.

### Sequencing data analysis

Perl scripts (available upon request) were developed to process the data from the RNA sequencing. Raw reads were first extracted from FASTQ files, and trimmed using a sequencing quality control of Q >13 [65]. Then the 3′ adaptor sequences within the read sequences were cleaned up. The prepared sequences were filtered and sequences with lengths ≥16 nt were aligned using Bowtie (version 0.12.8) [66] against both the human miRNA sequences downloaded from miRBase (Release 19, 2043 entries) [67] and the human genome reference sequences downloaded from the NCBI ftp site (Release 103). The Bowtie parameters that were used for the alignments were: -m 3 -n 1 -f -a --best --strata. Normalization of the miRNA profiles was based on the following formula:

(read counts of an individual miRNA/sum of read counts of all mappable miRNAs) multiplied by 1 × 10^6^.

The RNA sequencing data are available from the NCBI Gene Expression Omnibus database [GEO: GSE45722].

### Quantitative real-time PCR

To validate the RNA sequencing data, we performed a qPCR analysis of miR-92a-3p, miR-191-3p, miR26b-5p, and β-actin. The miRNA-specific miScript Primer Assays and the primer set specific for β-actin were purchased from QIAGEN (MS00006594 for miR-92a-3p, MS00031528 for miR-191-3p, MS00003234 for miR26b-5p, and QT01680476 for β-actin). First, 5 ng exosomal RNA or 20 ng cellular RNA was reverse transcribed by the miScript II RT kit (QIAGEN) at 37°C for 60 min, and then the enzyme was inactivated at 95°C for 5 min. After the activation of the polymerase enzyme at 95°C for 15 min, 40 cycles of 94°C for 15 s, 55°C for 30 s, and 72°C for 30 s were performed on the SteponePlus instrument (ABI). Melting curve analysis was used to confirm the specificity of the amplification reactions.

### Prediction of novel miRNA

To find novel miRNAs, we applied miRDeep2 and processed the raw sequencing data independently [47]. Predicted miRNAs with miRDeep2 total scores ≥2 were considered to be significant. If a predicted miRNA sequence resembled a reference rRNA or tRNA sequence, the sequence was discarded in the subsequent analysis regardless of the score.

### miRNA target gene enrichment analysis

We downloaded all miRNA target genes from miRDB (http://mirdb.org/miRDB/), an online database for miRNA target prediction and functional annotations. All the targets were predicted using MirTarget2 [68,69]. DAVID was used for the significant gene enrichment analysis. DAVID (Database for Annotation, Visualization and Integrated Discovery) (http://david.abcc.ncifcrf.gov/) provides a comprehensive set of functional annotation tools to understand biological meaning behind large list of genes [70]. Because each miRNA could target hundreds of genes, we limited the analysis to the top five most abundant exosomal miRNAs.

## Competing interests

The authors declare that they have no competing interests.

## Authors’ contributions

XH and LW performed the study design and drafted the manuscript. TY and ZS analyzed the data. XH, YL and ML constructed the sequencing libraries. MT and HJ performed the sequencing analysis. MK, SNT and LB provided blood plasma and edited the manuscript. MD, ML and RD edited the manuscript. All authors read and approved the final manuscript.

## Supplementary Material

Additional file 1 **Percentage of read counts with different insert sizes among the total mappable reads.** The NEBNext multiplex small RNA library preparation kit (NEB) generated more sequences with 21–23 nt inserts than did the other two kits that were tested. Overall represents the averages of the three different kits that were tested. Click here for file

Additional file 2 Read counts of the miRNAs detected in the 14 libraries (normalized to read number per million mappable miRNA seqeuences). Click here for file

Additional file 3 Top 20 RNAs in other RNA species (normalized to read number per million all mappable RNA seqeuences). Click here for file

Additional file 4 Putative miRNAs predicted by miRDeep2. Click here for file
