# Supplementary figures and images for: Characterization of human plasma-derived exosomal RNAs by deep sequencing
Source: BMC Genomics. 2013 May 10;14:319. doi: 10.1186/1471-2164-14-319 (PMC3653748; doi:10.1186/1471-2164-14-319)

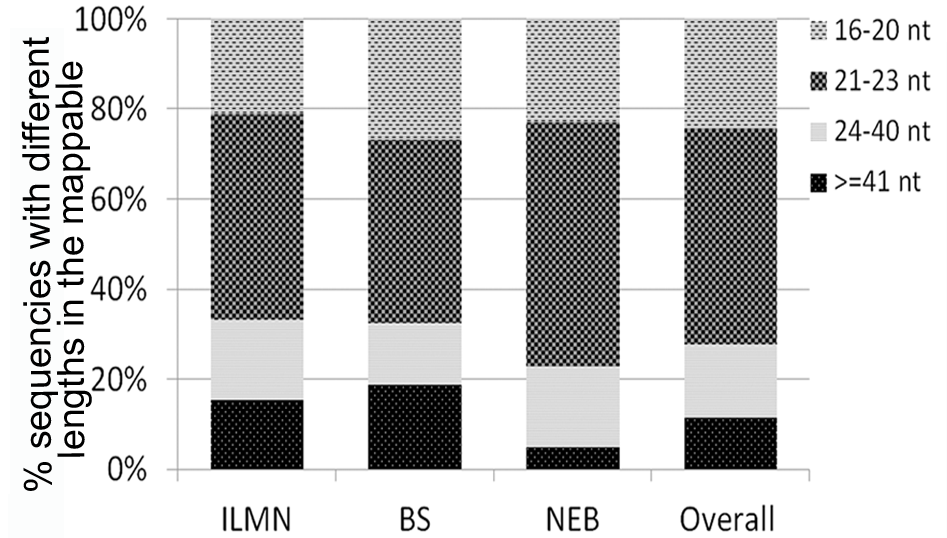

Supplement: Additional file 1 — Percentage of read counts with different insert sizes among the total mappable reads. The NEBNext multiplex small RNA library preparation kit (NEB) generated more sequences with 21–23 nt inserts than did the other two kits that were tested. Overall represents the averages of the three different kits that were tested. [file 1471-2164-14-319-S1.tiff]
